# Ether Anesthesia, Its History and Semi-Centennial Anniversary

**Published:** 1896-11

**Authors:** Nathan Jacobson

**Affiliations:** Syracuse, N. Y.; Professor of clinical surgery, College of Medicine, Syracuse University; surgeon to St. Joseph’s Hospital, 430 South Salina Street


### ETHER ANESTHESIA, ITS HISTORY AND SEMI-CENTENNIAL ANNIVERSARY.

By NATHAN JACOBSON, M. D., Syracuse, N. Y.,

Professor of clinical surgery, College of Medicine, Syracuse University; surgeon to St. Joseph's Hospital.

THE desirability of performing operations without pain was ever appreciated. As long ago as the I. Century of the Christian era, Herodotus wrote that the Scythians would intoxicate themselves by inhaling the vapor of hemp-seed, while Dioscorides and Pliny mentioned the anesthetic properties of mandrágora.

---

1. Read at the annual meeting of the Medical Association of Central New York, at Rochester, October 20, 1896.

In medieval times, the practice of narcotising patients with opium and hyoscyamus before operation, was in vogue, and it was thought necessary to reäwaken them by the administration of vinegar and fennel. It can be readily understood, however, that all of these attempts, though laudable, failed to accomplish their purpose.

At the beginning of the present century, Sir Humphrey Davy threw out the suggestion that "nitrous oxide may probably be used with advantage in surgical operations." His suggestion, however, failed of application, until a dentist, Dr. Horace Wells, of Hartford, Conn., witnessed the administration of the gas in the course of a popular lecture, given December 10, 1844, by Dr. G. Q. Colton, and was convinced of its practicability. Requesting Dr. Colton to administer the gas to him, he asked Dr. Riggs, a brother dentist, to extract a tooth while he might be unconscious. Recovering consciousness, Wells exclaimed: "A new era in tooth-pulling. It did not hurt me so much as the prick of a pin. It is the greatest discovery ever made." He continued its use in dentistry, as upwards of forty depositions of the best citizens of Hartford prove.

Feeling that the gas had a wider range than in the extraction of teeth, he visited Boston, where he had formerly practised dentistry, to induce the medical profession to use it in surgical operations. In January, 1845, a public attempt was made to render unconscious a patient, about to be operated upon by Dr. Warren, before the class at the Harvard Medical School. The experiment failed, as the unconsciousness was either too transient or not sufficiently profound.

The manufacture of ether, by the action of sulphuric acid upon alcohol, was described as early as the XIII. Century. A limited knowledge of the stimulating and benumbing qualities of the drug existed some time before its great usefulness was discovered, and these were even described in Pereira's Materia Medica in 1839. There are those among us who personally recall the "ether frolics" indulged in during the first half of the present century. It seems to have been a frequent practice at social gatherings, and particularly among college students, to inhale ether and enjoy the sport its intoxication would produce. I have been told by one who participated in these "frolics" that each of the participants would return home from these gatherings covered with bruises, whose origin he never could explain.

It was at the time that Dr. Wells was investigating the merits of nitrous oxide gas that Dr. E. E. Marcy, of Hartford, related to him that while a student at Amherst College he and his fellow-students had often inhaled both nitrous oxide and sulphuric ether, and, as far as he could determine, they seemed to act alike. Thus came the suggestion to use sulphuric ether to secure unconsciousness for operations.

The experiments of Drs. Marcy and Wells in this direction were evidently not satisfactory, for although the former succeeded in removing a small wen from a sailor without pain, still no attempt was made to extend its application or to induce the profession to adopt it.

At even an earlier date than this, likewise emanating from the use of ether at social gatherings for the purpose of provoking merriment, an incident occurred which was not made public until some fifteen years after the date of its occurrence. In the fall of 1839, at the home of a Mr. Ware, situated five miles from Athens, Ga., a colored boy was dragged into the house, where there had been a quilting party, and forcibly made to inhale ether. After a long struggle he became quiet, and even breathed stertorously. Fearful that they had committed murder, the young men who had administered the ether called a physician. The colored boy remained unconscious for an hour.

Dr. C. W. Long, of Jackson, Jefferson county, Ga., cognisant of the experiences of the "ether gatherings," made the first premeditated attempt to produce unconsciousness for the purpose of a surgical operation, on March 30, 1842, and removed a small glandular tumor from the neck of Mr. J. M. Venable. It was not until December, 1849, however, long after ether was being used by the profession generally, that Dr. Long saw fit to publish, in the *Southern Medical and Surgical Journal*, the result of his experience.

While Dr. Wells, to whose work with nitrous oxide reference has been made, was engaged in the practice of dentistry in the city of Boston, he had associated with him as a partner Dr. William T. G. Morton. The success of Dr. Wells with nitrous oxide induced Dr. Morton to attempt its use. At this time Dr. Morton, though a practising dentist, was engaged in the study of medicine. Not being himself competent to manufacture the gas, he applied to Dr. Jackson, a scientist of some repute, who then had a laboratory for analytical chemistry. Dr. Jackson asked him why he did not attempt to accomplish his purpose with sulphuric ether. Morton, acting upon the suggestion without further delay, improved the first opportunity, and on September 30, 1846, placed under the influence of ether a man by the name of Eben Frost, who presented himself at his office for the purpose of having a tooth extracted.

Prior to this time he had experimented with the drug upon his dog and other animals. Thoroughly impressed with the importance of this discovery he placed the matter before the surgeons of the Massachusetts General Hospital, asking permission to publicly demonstrate its serviceability in the performance of serious surgical operations.

On October 16, 1846, the first deliberate attempt in public at rendering unconscious a man about to be operated upon was made at the hospital. Perhaps the most vivid description of that, for surgery, epoch-making procedure is in the following words of Dr. Warren, the operating surgeon on that occasion :

The patient was arranged for the operation in a sitting posture and everything was made ready, but Dr. Morton did not appear for the lapse of half an hour. I was about to proceed when he entered hastily, excused himself for the delay, which had been occasioned by his modifying the apparatus for administration. The patient was then made to inhale a fluid from a tube connected with a glass globe. After four or five minutes he appeared to be asleep, and was thought by Dr. Morton to be in condition for operation. I made an incision between two and three inches long in the direction of the tumor, and to my great surprise, without any starting, crying out or other indication of pain. The fascia was then divided and the patient appeared wholly insensible. Then followed the insulation of the veins, during which he began to move his limbs, cry out and utter extraordinary expressions. These phenomena led to a doubt of the success of the application, and, in truth, I was not satisfied myself until I had, soon after the operation, and on various other occasions, asked the question whether he suffered pain. To this he always replied in the negative, adding, however, that he knew of the operation, and comparing the stroke of the knife to that of a blunt instrument passed roughly across his neck. Now that the effects of inhalation are better understood, this is placed in the class of imperfect etherisation.

It is of historical interest to note that at the "ether frolics" a sponge was used for the administration of the drug. The various appliances suggested for giving ether at operations were soon cast aside and again the sponge resorted to. In the Bigelow Operating

Amphitheater of the Massachusetts General Hospital, placed upon a glass saucer in one of the instrument cases, can be seen, still well preserved, the first sponge used for ether inhalation at operation.

A second application of ether was made on the following day, when Dr. Hayward removed a fatty tumor from the arm, the operation occupying about seven minutes, and the patient remaining entirely unconscious during its performance.

The surgeons refused, because of the concealment of the nature of the drug, to proceed further with its use. Dr. Morton proposed to patent the article and gave it the name of "letheon." With this in view he consulted R. E. Eddy, a lawyer. He learned from the attorney that inasmuch as Jackson had suggested the use of ether to him, a patent could be issued only to them jointly. The formal application read as follows :

STATE OF MASSACHUSETTS, }
COUNTY OF SUFFOLK. } SS.

On the 27th day of October, A. D. 1846, personally appeared before me Charles T. Jackson and William T. G. Morton, and made oath that they do verily believe themselves to be the original and first inventors of the improvement hereinbefore described, and that they do not know or believe the same to have ever before been known or used, and that they are citizens of the United States of America.

R. H. EDDY, J. P.

Finding that the surgeons were firm in their determination not to use "letheon" without receiving from him definite information as to its ingredients, Morton finally addressed a note to Dr. Warren, the surgeon-in-chief of the Massachusetts General Hospital, stating that it was merely sulphuric ether to which he had added certain volatile oils for the purpose of concealing its true nature. Its character known, it was again used used November 7, 1846, for the amputation of a thigh. This operation is thus described by the operator, Dr. Hayward :

The ether was administered by Dr. Morton. In a little more than three minutes she was brought under the influence of it ; the limb was removed and all of the vessels were tied but the last, which was the sixth, before she gave any indication of consciousness or suffering. She then groaned and cried out painfully. She afterwards said that she was wholly unconscious and insensible up to that time and seemed to be much surprised when told that her limb was off. She recovered rapidly, suffering less than patients usually do after amputation of the thigh, regained flesh and strength and was discharged well on the 22d of December.

Despite the fact that Dr. C. T. Jackson laid claim to being the original discoverer of the anesthetic qualities of ether, it was not until the 21st of November, 1846, that he witnessed for the first time an operation done under its influence. This was at the Massachusetts General Hospital, when a tumor was removed from the thigh.

Dr. Henry J. Bigelow, one of the surgeons of the hospital, who had interested himself much in this work, and who had at this time become so firmly convinced of the merits and safety of ether that he permitted his daughter to be etherised that she might have a tooth extracted painlessly, made, on the 3d of November, 1846, the first communication on the subject of ether anesthesia to the American Academy of Arts and Sciences. Six days later he presented a more extended paper to the Boston Medical Improvement Society. This paper was published in the *Boston Medical and Surgical Journal*, November 18, 1846, and was the first announcement to the medical world of this important contribution to surgical science.

Within six weeks Europe was aroused and the value of ether anesthesia was attested by the surgeons of London, of whom Liston was the first to resort to its use. January 2, 1847, there appeared a letter addressed by him to a Dr. Boott, in which he says:

> I tried ether inhalation today in a case of amputation of the thigh, and in another requiring an evulsion of both sides of the great toe-nail, one of the most painful operations in surgery, and with most perfect and satisfactory results. It is a very great matter to be able to thus destroy sensibility to such an extent and without apparently any bad result. It is a fine thing for operating surgeons, and I thank you most sincerely for the early information you were so kind to give me of it.

A little later its use was general in the Parisian hospitals. On the other hand, its adoption in this country was much more tardy. Several weeks elapsed after the appearance of Bigelow's paper before any notice whatsoever was taken of it by the medical press of the United States. Then the expressions were those of doubt and incredulity. The first record I find of an operation performed in New York City was February 13, 1847, when a thigh was amputated for chronic synovitis. In Philadelphia the first operation seems to have been done at the Jefferson Medical College,

July 19, 1847, for the resection of a conical stump. These dates are taken from a table, which accompanies the report of the committee on surgery of the American Medical Association, and appear in Volume I. of the transactions of that society.

The delay in the acceptance of ether for anesthetic purposes can be readily understood by you, cognisant, as you are, of the strong opposition the medical fraternity has always manifested to patented articles. The patent applied for was granted on the ground that although the drug had long been known, and that there was a general knowledge of its character, no one had ever attempted, as far as was then known, to apply it for the purpose of producing insensibility to pain in surgical operations. It was at that time customary to sell office rights, permitting the use of patented articles, and Morton proceeded at once to restrict the use of ether for surgical purposes to those who would purchase of him the privilege. Litigation followed, and the government ultimately failed to recognise the validity of the patent. In the meantime, however, the most bitter opposition had been awakened.

Some of the medical journals had seen fit to characterise in the strongest terms the course of the Boston surgeons in consenting to bolster up a patented article. As voicing the sentiment of many of the profession, and in explanation of the slow progress made in establishing ether anesthesia in the United States, I would refer to an editorial, from which I make a few extracts, which appeared in the issue of the *Medical Examiner* for December, 1846. Its editor, Robert M. Huston, was at that time one of the professors in the Jefferson Medical College. Amongst other things he says:

> Looking upon this as nothing more nor less than a new scheme to tax the pockets of the enlightened public, we should not consider it entitled to the least notice, but that we perceive by the *Boston Medical and Surgical Journal*, that prominent members of the profession in that city have been caught in its meshes. . . . .

Then follows a statement of the reasons given by the Boston surgeons for using ether and a description of the manner of its administration. When the editor again proceeds, it is after this fashion:

> We are persuaded that the surgeons of Philadelphia will not be seduced from the high professional path of duty into the quagmire of quackery by this will-o'-the-wisp, and if any of our respectable dentists should be tempted to try this new patent medicine, we advise them to consider how great must be the influence of an agent on the nervous system to render a person unconscious to pain—the danger there must necessarily be from such overpowering medication, and that if a fatal result should happen to one of their patients, what would be the effect upon their conscience, their reputation and business, and how the practice would be likely to be viewed by a Philadelphia court and jury ?

We cannot close these remarks without again expressing our deep mortification and regret that the eminent men who have so long adorned the profession in Boston, should have consented for a moment to set so bad an example to their younger brethren as we conceive them to have done in this instance. If such things are to be sanctioned by the profession, there is little need of reform conventions or any other efforts to elevate the professional character. Physicians and quacks will soon constitute one fraternity.

But the continued favorable reports that appeared both abroad and in the Boston journal helped enormously to displace the prejudice that had been created. Prof. Simpson, who the next year introduced chloroform as an anesthetic in obstetric cases, was able at the end of this year to state that 300 important surgical operations had been performed under ether, the reports of which he had collected from the different hospitals in England, Ireland and France, and that the death-rate had been smaller than before the introduction of anesthesia.

Three and one-half years after the introduction of ether, Dr. Warren reported that there had been over 1,000 etherisations in the Massachusetts General Hospital, and many more in private practice in the City of Boston, without a single death, and that no one had seen fit to relinquish it, while the circle of those using it was constantly increasing.

The introduction of chloroform by Simpson aided largely in favorably establishing anesthesia abroad. It is unnecessary to raise the question at this time as to whether chloroform was an American discovery by Dr. Samuel Guthrie, of Sackett's Harbor, N. Y., or whether Professor David Waldie, Master of Apothecaries Hall, Edinburgh, had suggested the extraction of chloroform from chloric ether to Simpson. Nobody will attempt to dim the luster which illumines the name of Simpson nor in the least detract from the glory of his great discovery.

It is most unfortunate, on the other hand, that the desire for personal gain and fame should have created what might properly be called an internecine war amongst those so closely associated with the discovery of anesthesia in America. Wells, with whom it will be remembered Morton was associated in the practice of dentistry, went abroad for the purpose of establishing his claim to the discovery of anesthesia by the inhalation of vapors. He failed to make the impression that he had hoped. Upon his return to this country, he found that his former partner and Dr. Jackson were being recognised as the discoverers of a far more successful method, and deep mental gloom fell upon him. The victim of melancholia, in a mad fit of frenzy, he was arrested in New York City for having thrown vitriol upon some ladies, and while insane committed suicide in one of the prisons of New York City.

Jackson, who did not at first appear as the discoverer of ether anesthesia, after its success was assured in this country, despite his lack of active coöperation in its introduction, forwarded to the Academy of Science in Paris, to be placed in their archives, two sealed communications, which, some weeks after, were opened at his request by his friend, M. Elie de Beaumont, at a meeting of that society, January 18, 1847. In these communications Jackson formally announced to the French Academy that he had discovered the value of the administration of ether vapor in surgical operations and that all that Morton had done had been under his direction. The great secrecy which had been maintained, Velpeau remarked in discussing these communications, was without reason, as he had received personal letters from Dr. Warren, of Boston, communicating the same information a month prior.

Malgaigne had succeeded better than any other surgeon at that time in Paris with ether. At the same meeting other surgeons recorded their experiences, and while a conservative tone marked the proceedings of the meeting, it was evident that ether had come to stay in France, although, commenting upon it, one of the editors of the day used this expression: "Before twelve months are completed, many shall have recovered from this etherising revery and the subject will then be considered in its true light and on its actual merits."

The struggle between Morton and Jackson as to who deserved the more credit grew with each year more fierce, and had upon each of these men a most depressing influence. The matter was carried to congress, and that body was memorialised to adequately recognise the services rendered by Morton. The committee to whom the matter was entrusted finally reported that Dr. Horace Wells did not make any discovery as to the value of the vapor of sulphuric ether, which he considered reliable; that Dr. Jackson did not appear at any time to have discovered any use of ether which had not appeared in print some years before in Great

Britain; that Dr. Morton, in 1846, did discover that ether would prevent pain in surgical operations; that it might be given for that purpose without danger to life and demonstrated its applicability and importance in capital operations; that Dr. Jackson had the belief that ether would accomplish this, and had advised various persons to attempt its application, but that neither they nor he took any steps to accomplish this end, and not until Dr. Morton's experiments was this made a certainty. Despite this report our government made no substantial recognition of Morton's services. England gave Sir William Jenner the sum of $225,000 in recognition of the discovery of vaccination. But a year or two ago France decorated and presented a large sum to Roux for his work with diphtheria antitoxin, although no pretense can be made that he was even its discoverer.

Disheartened and impoverished, Morton went from city to city asking and accepting such gifts as the medical profession and the public generally might see fit to give him. The Massachusetts medical profession, however, expressed its appreciation by presenting him with a small silver casket containing $1,000. Both Morton and Jackson, however, received recognition from foreign societies, and had bestowed upon them various decorations, and each received from the French Academy 2,500 francs. The city of Hartford, loyal to her citizen, erected a monument which presents a portrait statue of Dr. Wells, and bears the inscription, "Horace Wells, who discovered anesthesia, November, 1844."

In the Public Garden of the City of Boston is a stately monument, dedicated in June, 1868. It is of granite and red marble, and is surmounted by a group illustrating the story of the Good Samaritan. On the face of the pedestal, beneath, are the words, "To commemorate the discovery that the inhaling of ether causes insensibility to pain. First proved to the world in Boston, October 16, 1846." On the reverse side appear the words, "The gift of Thomas Lee," and the inscription, "In gratitude for the relief of human suffering, a citizen of Boston had erected this monument, A. D. 1867." The name of neither Morton or Jackson is inscribed hereon. The word "anesthesia" is said to be the coining of Oliver Wendell Holmes, and our witty poet doctor, when asked whose name should be placed upon this monument, Morton or Jackson, said, "Oh, call it either," and so this is known as the "ether monument." The new City Library of Boston has surrounding its upper story a series of memorial tablets bearing the names of perhaps 500 men, famous in history, literature and science, and upon one of them we find inscribed this group, "Astley, Cooper, Warren, Jackson, Morton, Lister."

The failure to recognise definitely either Morton or Jackson as the discoverer of anesthesia, preyed upon the minds of both of these men. Jackson became mentally unbalanced and died in an insane asylum, at Summerville, Mass., August 20, 1880. In the September, 1896, number of *McClure's Magazine*, the wife of Dr. Morton has presented in a beautiful way, which speaks for her devotion to her deceased husband, the story of his struggles, and pictures most feelingly the end of her husband. He, with nerves shattered, really demented, sprang from his carriage as they were driving through the upper end of Central Park, soon became unconscious, and died before he could reach St. Luke's hospital, whither an effort was made to take him.

In Mt. Auburn cemetery, where are buried Holmes, Longfellow, Lowell, Agassiz, Sumner and a host of eminent men, I stood recently beside a grass plot some eighteen feet square, enclosed in a rounded stone coping, where the remains of Dr. Morton rest. Upon the four sides of the base of a modest marble monument these inscriptions can be found: "Erected by the citizens of Boston." "William T. G. Morton, inventor and revealer of anesthetic inhalation, born August 19, 1819, died July 15, 1868." "Before whom in all time surgery was agony." "By whom pain in surgery was averted and annulled." "Since whom science has control of pain."

A half century has passed since Morton established the value of the anesthetic inhalation of ether. The history of surgery during this period marks an era of progress unequaled by all the ages that preceded it. Reviewing the surgical operations performed during the time which antedated the introduction of ether, it is, indeed, a matter of great surprise to find that capital operations of the most serious character on all the vital organs of the body have been successfully performed.

The surgeon of the pre-anesthetic era must have been a man possessing qualities today no longer considered necessary. He must have been a man of great decision, unerring celerity, bold and fearless. He must have possessed manual dexterity, which challenged the admiration of the medical observer. He must have been firm, even to a degree of coldness. True, operations could not have been performed with the same deliberation, nor could the technique have been as complicated nor as carefully arranged in its details as today.

Under the influence of anesthesia the field of operation immediately widened. To the patient the operating room had been a chamber of horrors ; the agony to be suffered must have instilled those requiring surgical attention with deep fear and deterred many from yielding to the demands of the surgeon, even where comfort could be secured or life saved. Robbed of its terror the patient submitted to operation, and at once not only were the established operations more frequently performed, but new surgical procedures undertaken. Look where we will, we find this statement amply verified.

Bone surgery received new impulse. Ununited fractures were subjected to drilling ; excisions of various bones were performed. Resection of the ribs to give exit to pus collections in the chest, and excision of the hip and other joints were successfully performed for the first time. Vessels, which never before had been, were now ligated. As operations could be undertaken with deliberation, careful dissections were made, and time was no longer an element in their performance. Anesthesia rendering the patient absolutely submissive, the surgeon could now proceed without fear, as Mott described, "that the writhings of the sufferer should convert him who sought to be the deliverer into the executioner." The innominate, the vertebral, the primitive and two internal carotid simultaneously, the subclavian in its first portion, the primitive iliac, the two internal iliacs simultaneously, were all ligated for the first time successfully after the introduction of ether.

What will we say of the great cavities of the body ? Anesthesia made the application of antisepsis and asepsis possible. Through the aid of these two great benefactions, the territory of the surgeon recognises no limit. Even as early as 1855 the lateral ventricle was opened for the purpose of evacuating pus, while now the invasion of the skull has been undertaken so frequently and so extensively that surgical art has been applied to almost every known pathologic condition occurring in the head. Neither has there been any limit to the invasion of the abdominal cavity, the mere opening of which has come to be regarded as an innocent procedure. From being undertaken only occasionally and in extreme cases, laparatomy, attended with a death-rate of almost 50 per cent., is performed for every possible pathologic condition encountered in the abdomen, and except in conditions of great gravity, without fear as to the outcome.

Intestinal surgery was substantially unknown fifty years ago. The treatment of the septic conditions within the abdomen is entirely a modern creation. The gall-bladder, the liver, the kidneys, the spleen, the pancreas, have all been laid under the surgeon's knife.

Since gastrotomy was first performed, in 1849, the stomach has been regarded as a legitimate field for surgical invasion, and despite the fact that, in the main, the surgery of this organ, especially for the relief of malignant disease, has not been eminently successful, without doubt, much misery has been relieved and many times death averted. The urinary bladder has been treated for its various surgical conditions by the operator with success almost unparalleled. Since Bigelow transformed, through the agency of the anesthetic, at whose birth he was present, the operation of lithotrity into that of litholopaxy, the crushing of stone has indeed been quite a different and much more successful operation. Plastic and conservative surgery are new arts.

But there is no occasion for me to weary you by presenting a further list of operative procedures made possible by anesthesia. This is not a theme new to you. Not alone, however, were new operations made possible ; the results were improved, both because patients submitted to operation earlier and they could be better performed. More than this, it was by no means unheard of that patients died because of the tremendous shock attending painful operations, when the suffering was unrelieved.

A fortunate occurrence attended the first proposed attempt to use chloroform. Simpson had arranged to administer it in a surgical operation. Being deterred, it had to proceed without the anesthetic. The patient died upon the table. It would have been a sad blow for chloroform had such a result attended its first exhibition.

The average mortality in Guy's Hospital in strangulated hernia up to 1842 was 50 to 61 per cent., and at Wurzburg, up to 1846, 48 to 52 per cent. Of 275 cases of amputation of the arm, collected in 1853, 103 had died. In amputation of the lower extremities, performed in Great Britain in civil practice between 1834 and 1845, there was one death in three, while in America, one in four. These figures might be multiplied, but their mere mention is sufficient to indicate what a great change has been wrought since the introduction of ether.

Nor is this all. In the recognition and reduction of fractures and dislocations, ether has proven of lasting benefit. As opposed to chloroform, the great value of ether stands out in the stimulation it produces, permitting long-continued surgical operations without marked depression. Anesthesia, furthermore, has assisted surgical diagnosis to a marked degree. Not only has it been possible to examine the most sensitive organs, to explore them, if necessary, with instruments, to relax muscular tension, to permit free palpation, but also, where necessary, exploratory operations can be undertaken.

Instead of stupefaction through narcotic drugs, or alcoholic intoxication, carried to partial anesthesia, instead of depending upon the celerity of the operator or section of the nerves, ether came at once to be recognised as an agent capable of securing freedom from pain and rendering the patient unconscious of the surgeon's attack. True, at first the most absurd claims were made and had to be disproven. Every unfortunate result attending an operation was attributed to its use. But so overwhelming was the evidence in favor of it, that in less than a year's time it was recognised as the greatest gift medicine had ever presented to mankind. As we look back over the glorious march surgery has made since its introduction, we cannot help but confirm the predictions made that anesthesia would be the greatest triumph of the century.

In the face of the opposition to etherisation, both on the part of the medical press and medical men of our country, growing out of the unfortunate manner proposed to secure its introduction into general use and against the selfish aims of its discoverers, in what bold relief do the eminent surgeons of the Massachusetts General Hospital stand out. Warren, Bigelow and Hayward speedily recognised the intrinsic worth of Morton's discovery. Convinced of the value of ether for the purposes claimed, they were ready to give it a trial without knowing its true character. But having recognised its value, for the benefit of science, as well as for the protection of mankind, they positively refused to further proceed with it until its true nature was revealed to them. When this was made known, without any delay whatsoever, they spread before the medical world their experience. To their enthusiastic support, in the face of the most bitter opposition, more than to aught else, do we owe its introduction. Long before they had aroused the medical profession on this side of the Atlantic to a full appreciation of ether, the response came from abroad in words of loudest praise, from the surgeons of London and Paris.

And yet when we consider what a priceless boon anesthesia has proven, how full of pathos is the end of those most closely connected with its origin. Wells, Jackson and Morton, each anxious to be recognised as the author of this great blessing, seeking through it fame and fortune, not only failed to gain either, but steeped in melancholy, mental wrecks, passed out of the world almost unrecognised.

Surely so great an achievement is deserving of the everlasting gratitude of all mankind. No monument can be too grand for its commemoration, no recognition commensurate with its beneficence.

430 South Salina Street.

